# Plant-Produced Anti-Enterovirus 71 (EV71) Monoclonal Antibody Efficiently Protects Mice Against EV71 Infection

**DOI:** 10.3390/plants8120560

**Published:** 2019-12-01

**Authors:** Kaewta Rattanapisit, Zhang Chao, Konlavat Siriwattananon, Zhong Huang, Waranyoo Phoolcharoen

**Affiliations:** 1 Research Unit for Plant-Produced Pharmaceuticals, Chulalongkorn University, Bangkok 10330, Thailand; 2 Vaccine Research Center, CAS Key Laboratory of Molecular Virology & Immunology, Institut Pasteur of Shanghai, Chinese Academy of Sciences, Shanghai 200031, China; chaozhang@ips.ac.cn; 3 Research Unit for Plant-Produced Pharmaceuticals and Department of Pharmacognosy and Pharmaceutical Botany, Faculty of Pharmaceutical Sciences, Chulalongkorn University, Bangkok 10330, Thailand

**Keywords:** enterovirus 71 (EV71), hand-foot-mouth disease, plant biotechnology, monoclonal antibody, molecular pharming, plant-produced monoclonal antibody, *Nicotiana benthamiana*

## Abstract

Enterovirus 71 (EV71) is the main causative agent of severe hand-foot-mouth disease. EV71 affects countries mainly in the Asia-Pacific region, which makes it unattractive for pharmaceutical companies to develop drugs or vaccine to combat EV71 infection. However, development of these drugs and vaccines is vital to protect younger generations. This study aims to develop a specific monoclonal antibody (mAb) to EV71 using a plant platform, which is a cost-effective and scalable production technology. A previous report showed that D5, a murine anti-EV71 mAb, binds to VP1 protein of EV71, potently neutralizes EV71 in vitro, and effectively protects mice against EV71 infection. Herein, plant-produced chimeric D5 (cD5) mAb, variable regions of murine D5 antibody linked with constant regions of human IgG1, was transiently expressed in *Nicotiana benthamiana* using geminiviral vectors. The antibody was expressed at high levels within six days of infiltration. Plant-produced cD5 retained its in vitro high-affinity binding and neutralizing activity against EV71. Furthermore, a single dose (10 µg/g body weight) of plant-produced cD5 mAb offered 100% protection against infection in mice after a lethal EV71 challenge. Therefore, our results showed that plant-produced anti-EV71 mAb is an effective, safe, and affordable therapeutic option against EV71 infection.

## 1. Introduction

Hand-foot-mouth disease (HFMD) mainly affects children under five-years-old. HFMD is of considerable public health concern in the Asia-Pacific region because of the economic burden in the affected countries. In addition to hospital expenditure, loss of time from work is an additional cost, because families have to care for the sick children away from school [1]. Considering the economic burden of HFMD, the development of HFMD vaccine and a therapeutic drug is required urgently.

HFMD is caused by several viruses such as enterovirus 71 (EV71) and coxsackieviruses [2]. EV71 causes severe central nervous system or cardiovascular complications, leading to the death of infected patients [3,4]. Various types of EV71 vaccines have been developed previously, such as inactivated whole-virus vaccines [5,6,7,8,9], live attenuated vaccines [10,11], recombinant vaccines [12,13,14,15], and peptide vaccines [16,17]. For EV71 post-exposure treatment in patients with severe infection, EV71-specific intravenous immunoglobulins are used [18]. However, there are disadvantages to using pooled human sera, such as the risk of transmitting human pathogens, availability of donors, and batch-to-batch variability [19]. Therefore, neutralizing monoclonal antibodies (mAbs) are considered a safe alternative for passive immunization against EV71.

Over the years, several EV71-neutralizing antibodies have been reported [20,21,22,23,24,25,26]. Among these mAbs, D5 is one of the most promising mAb for development as prophylactic and therapeutic for EV71 infection. D5 mAb binds specifically to SP70 peptide on VP1 GH loop of EV71 and can potently neutralize EV71 infection by blocking viral attachment and internalization and by stabilizing the virus through a bivalent binding mode [26,27]. However, the antibody production in mammalian cell culture has limitations with high production costs and scalability.

Recently, plants are being used as an alternative mAb production platform with unique advantages such as low production cost, speed of production, and scalability [28]. This platform offers a solution for developing countries to develop affordable medicines in the region. Previous studies demonstrated the efficacy of plant-produced mAbs for viral infections, such as West Nile virus [29], Ebola virus [30], Rabies virus [31], and others. The goal of this study is to investigate the protective effect of plant-produced EV71 specific mAb against EV71 challenge in mice.

In this study, chimeric D5 (cD5) mAb was constructed and produced transiently in *Nicotiana benthamiana* leaves. After protein-A affinity chromatography, the purified antibody was tested for binding, neutralization, and protection against EV71 infection. The plant-produced cD5 mAb bound to its epitope, SP70, and effectively neutralized EV71 and protected mice against EV71 lethal challenge. Our data confirmed that the plant platform is a promising antibody production system for producing effective mAbs for the post-exposure treatment of EV71 infection.

## 2. Results

### 2.1. Expression of cD5 mAb in Nicotiana benthamiana

**Figure 1:**
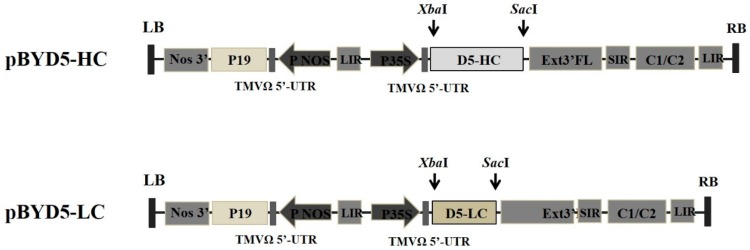
Constructs of chimeric D5 (cD5) heavy chain and light chain genes in geminiviral vectors used in the study. P35S: Cauliflower Mosaic Virus (CaMV) 35S promoter, TMVΩ 5′-UTR: 5′ untranslated region of tobacco mosaic virus Ω, D5-HC: heavy chain of cD5 antibody, D5-LC: light chain of cD5 antibody, Ext3′FL: 3′ full length of tobacco tabacum extension gene, SIR: short intergenic region of BeYDV genome, LIR: long intergenic region of BeYDV genome, C2/C1: Bean Yellow Dwarf Virus (BeYDV) ORFs C1 and C2 which encode for replication initiation protein (Rep) and RepA, PNOS: nopaline synthase promoter, P19: P19 gene from Tomato Bushy Stunt Virus (TBSV), Nos3′: 3’ termini of the polyadenylated nos mRNA.

**Figure 2:**
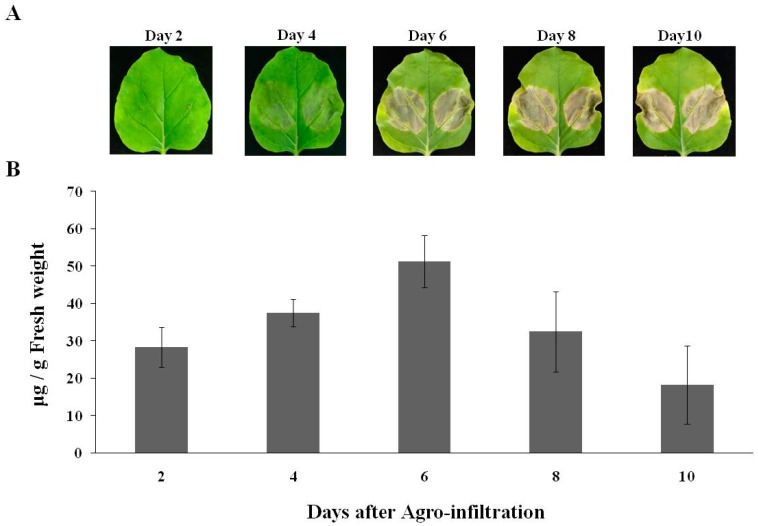
Day optimization of cD5mAb expression in *N*. *benthamiana* leaves. Quantification of plant-produced cD5 mAb was determined on day 2, 4, 6, 8, and 10 after agroinfiltration using ELISA. The leaf necrosis (**A**) and yield of cD5 mAb (**B**) were shown. Data are means ± SD of triplicates.

To construct chimeric mAb, variable regions of murine D5 antibody were linked with constant regions of human IgG1 heavy chain (HC) and kappa light chain (LC) and then separately cloned into a geminiviral vector, resulting in pBYD5-HC and pBYD5-LC (Figure 1). *N. benthamiana* leaves were agro-coinfiltrated with both pBYD5-HC and pBYD5-LC. In a time-course experiment, leaf samples were harvested 2-, 4-, 6-, and 8-days after infiltration. The leaves infiltrated with pBYD5-HC and pBYD5-LC showed necrosis on the sixth day after infiltration (Figure 2A). The amount of chimeric D5 (cD5) mAb expressed in *N. benthamiana* leaves was measured using ELISA. The optimal harvest time is six days after infiltration, and the expression level of cD5 mAb is 50 µg/g of fresh leaf weight (Figure 2B).

### 2.2. Purification of cD5 mAb from N. benthamiana Leaves

**Figure 3:**
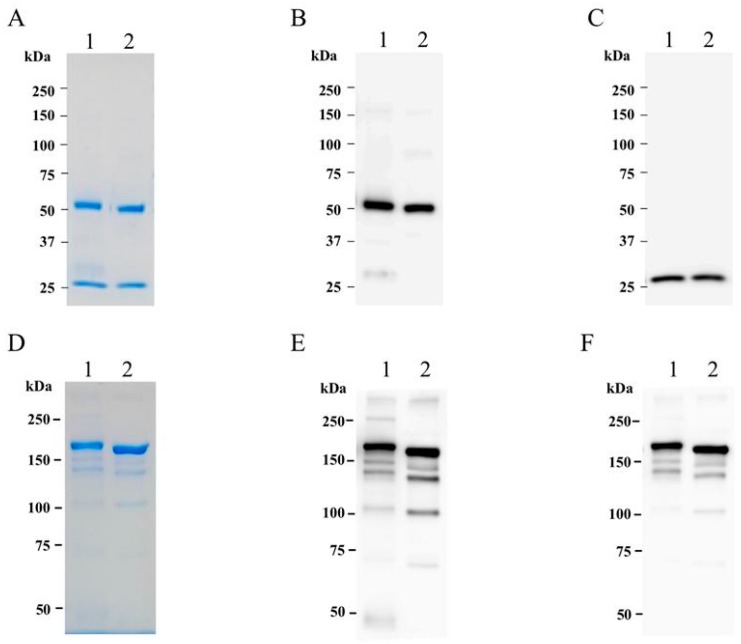
SDS-PAGE and western blot analysis of purified plant-produced cD5 mAb. Purified plant-produced cD5 mAb (lane1) and irrelevant human IgG (lane2). Panels (**A**), (**B**), and (**C**) show SDS-PAGE gradient gel (6–15%) results of the antibodies under reducing condition with InstantBlue™, anti-Gamma, and anti-Kappa, respectively. Panels (**D**), (**E**), and (**F**) showed results of 6% SDS-PAGE gel results of the antibodies under the non-reducing condition with InstantBlue™, anti-Gamma, and anti-Kappa, respectively.

A simple, standard protein-A affinity chromatography was used to purify cD5 mAb from N. benthamiana leaves. After the extraction process, plant crude proteins were filtered using 0.45 µm filters and separated by protein-A chromatography. The purified cD5 protein sample was characterized by SDS-PAGE and western blot analysis. Under reducing condition, the heavy chain and light chain of cD5 mAb bands were present at 50 kDa and 25 kDa, respectively (Figure 3A). Western blot analysis with anti-human gamma and anti-human kappa antisera confirmed the expression of heavy and light chain, respectively (Figure 3B,C). Under the non-reducing condition, the purified cD5 mAb band was visible at 150 kDa (Figure 3D), confirming the assembly of the whole IgG molecule (two heavy chains and two light chains). Western blot analysis with anti-human IgG and anti-human kappa antisera confirmed the expression of the heavy chain and light chain in the whole IgG molecule (Figure 3E,F). The purified cD5 mAb was used for further in vitro and in vivo studies.

### 2.3. Plant-Produced cD5 mAb Retains Antigen-Binding Activity

**Figure 4:**
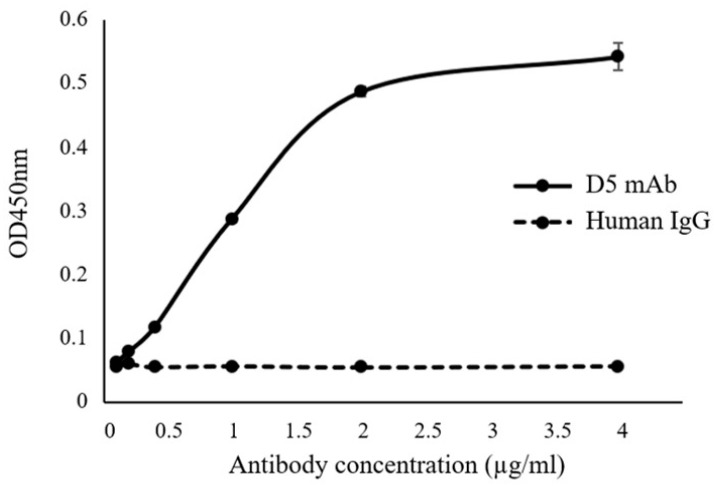
Determination of binding affinity of plant-produced cD5 mAb to synthetic SP70 peptide by ELISA. Synthetic SP70 peptide was used as the coating antigen and irrelevant human IgG was used as a negative control. The representative points are the mean values of triplicate assays conducted at each concentration of the antibodies.

The purified plant-produced cD5 mAb was tested for binding in ELISA using synthetic SP70 peptide. Plant-produced cD5 mAb or human IgG control antibody (Abcam, Cambridge, UK) was serially diluted before incubation with immobilized SP70 peptide. The binding was detected by horseradish peroxidase (HRP) labeled anti-human IgG antiserum at an absorbance of 450 nm (OD450). As shown in Figure 4, plant-produced cD5 mAb showed saturable dose-dependent binding to SP70 peptide. By contrast, IgG control antibody did not show any binding activity, regardless of antibody dose. These results indicate that plant-expressed cD5 mAb has the ability to bind to the SP70 epitope recognized by murine D5.

### 2.4. Plant-Produced cD5 mAb Efficiently Neutralized EV71

**Figure 5:**
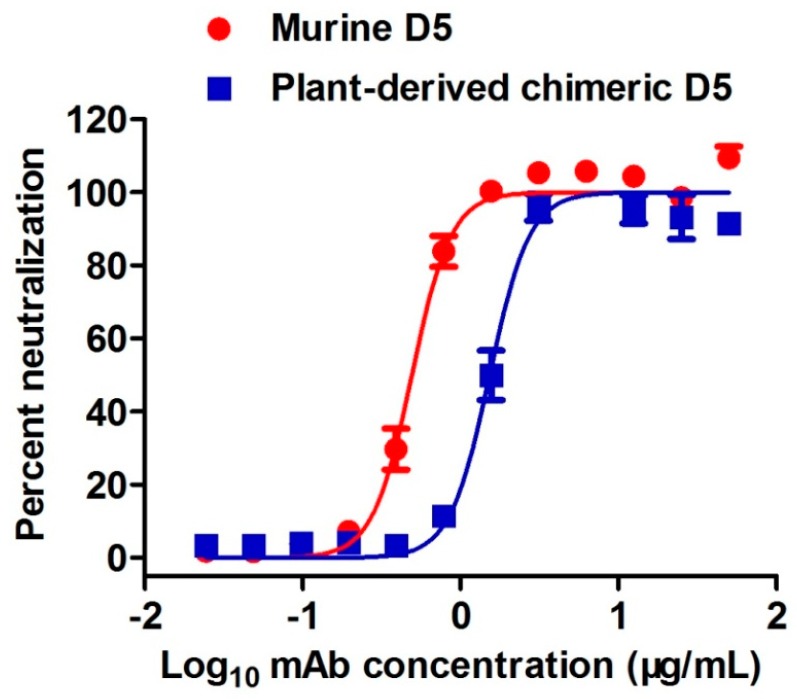
Neutralizing activity of plant-produced cD5 mAb against EV71. One hundred TCID50 of EV71 strain EV71/G082 was mixed with serial two-fold dilutions of plant-produced cD5 or murine D5 antibodies and incubated for 1 h. The mixtures were added to rhabdomyosarcoma cells (RD cells) and incubated for three days. Cell viability was measured, and results were expressed as percent neutralization. Data are expressed as mean ± SEM of five replicate wells.

Plant-produced cD5 mAb was tested in vitro for its ability to inhibit EV71 infection using micro-neutralization assay. Murine D5 antibody served as the positive control in this assay. Figure 5 shows a dose-dependent inhibitory effect of plant-produced cD5 mAb with an IC50 value of 1.53 µg/mL. Moreover, there was no significant difference (*p* = 0.2844; *p* > 0.05) in the neutralization capacity between the plant-produced cD5 mAb and murine D5 antibody (IC50 value = 0.49 µg/mL). These results showed that plant-derived cD5 mAb retains the neutralizing activity of murine D5 mAb.

### 2.5. Plant-Produced cD5 mAb Protected Recipient Mice Against Lethal EV71 Infection

**Figure 6:**
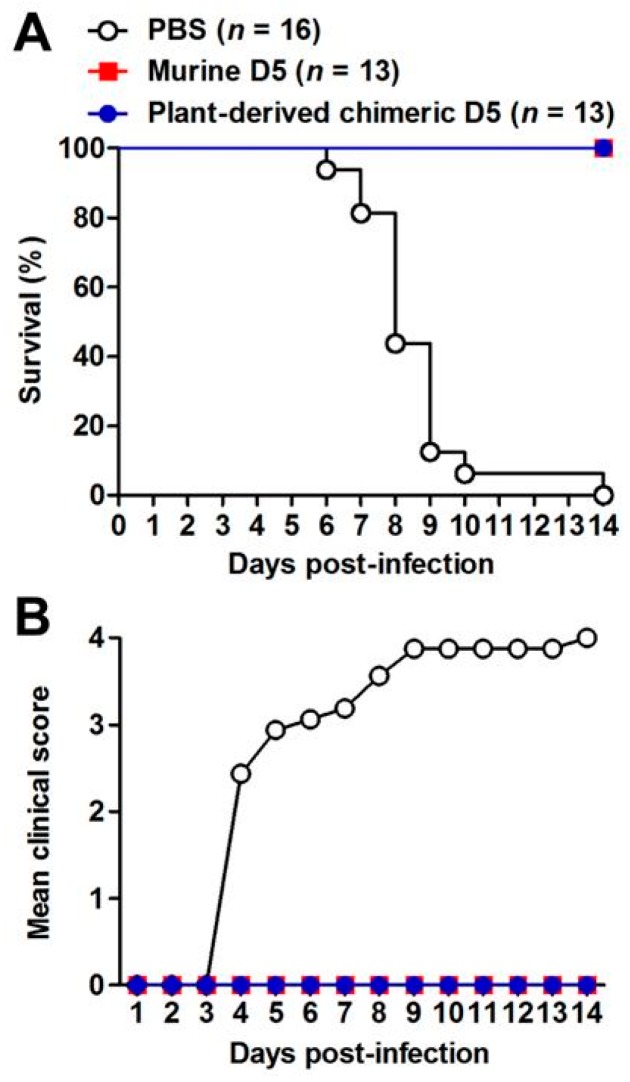
Plant-derived cD5 antibody fully protected mice against EV71 infection. Groups of 5-day-old ICR mice were infected with EV71/MAV-W, and 24 h later injected with phosphate-buffered saline (PBS), plant-produced cD5 antibody, or murine D5 antibody. The infected mice were monitored daily for 14 days for (**A**) survival and (**B**) clinical score. Clinical scores were graded as follows: 0, healthy; 1, reduced mobility; 2, limb weakness; 3, paralysis; and 4, death. The number of mice in each group was indicated in the bracket.

The therapeutic efficacy of plant-produced cD5 mAb was assessed in a neonatal mouse model of EV71 infection, based on the mouse-adapted EV71 strain, EV71/MAV-W. Groups of 5-day-old ICR mice were infected with a lethal dose of EV71/MAV-W. Twenty-four hours later, a single dose of PBS, 10 µg/g of plant-produced cD5 mAb, or 10 µg/g of murine D5 mAb were administered to mice. Note that in this experiment, murine D5 mAb and PBS were used as positive and negative controls, respectively. After infection, mice in the PBS group developed signs of paralysis gradually and died eventually within 14 days post-infection. In contrast, all mice treated with plant-produced or murine D5 antibodies remained symptom-free, and all of them survived (Figure 6). These results indicated that plant-produced cD5 mAb could protect mice against lethal EV71 infection.

## 3. Discussion

Despite the severe enterovirus 71 (EV71) outbreaks in Asia, currently, there is no effective treatment [32]. The industrial standard for therapeutic antibody production is the use of mammalian cells [33]. However, the high production cost and limited scalability limit availability of these antibodies for therapeutic use in regions with HFMD outbreak. Several advantages of production of mAbs using the plant platform encouraged the possible utility of this platform in the developing countries, due to low production cost, rapid production, lack of animal and human pathogens, and scalability [34,35,36]. Several mAbs produced in plants are in clinical trials for tooth decay [37], HIV [38], and cancer [39]. Here, we investigated the possibility of producing a therapeutic mAb against EV71 in plants.

There are several mAbs specific to EV71, for example, mAb specific to RNA-dependent RNA polymerase [40], mAb binding to EV71 VP1 [20,22,24,25,26], mAb binding to EV71 VP2 [41], mAb binding to VP3 [42], and others. Among these mAbs, D5 is one of the most potent anti-EV71 neutralizing mAb. It is specific to the highly conserved VP1 GH-loop of EV71 and was shown to have a broad cross-neutralization activity against all tested EV71 strains [26,27,43]. Therefore, this mAb has the potential to be an effective treatment for EV71 infection. In the present study, we report the transient expression of chimeric D5 (cD5) mAb in a plant system.

In this study, murine D5 antibody was engineered into a chimeric antibody cD5 to increase the clinical potential of this antibody. The cD5 mAb was expressed at high levels in *N. benthamiana* leaves within six days after agroinfiltration (Figure 2) and efficiently assembled into a native IgG molecule (Figure 3). With the codon-optimization, cD5 mAb accumulated at an average of 50 µg/g fresh leaf weight (Figure 2). The results showed that plant-produced cD5 mAb could bind to SP 70 peptide of the VP1 on EV71 (Figure 4) and efficiently neutralize EV71 in vitro (Figure 5), demonstrating that plant-derived cD5 mAb retains the binding and neutralizing activities of murine D5 mAb.

To be considered a therapeutic agent, the plant-made cD5 mAb must have in vivo efficacy. The mouse-adapted EV71 infection in ICR mice caused lethal infection. In this study, the plant-produced cD5 mAb protected all mice from EV71 challenge, similar to murine D5 mAb used as a positive control. To our knowledge, this is the first report on the plant-produced mAb showing successful protection of mice against EV71 lethal challenge. Our in vivo results showed the potential of plant-produced cD5 mAb as an efficacious post-exposure treatment. In conclusion, plant-produced cD5 mAb has the potential to be developed further for use as EV71 diagnostic and post-exposure prophylactic agent during HFMD outbreaks.

## 4. Materials and Methods

### 4.1. Gene Construct

The variable region gene sequences of D5 heavy chain (VH) and light chain (VL) were codon-optimized for using GeneArt and synthesized. To construct heavy chain of cD5 antibody, the VH gene and constant region of human IgG1 heavy chain (CH) was cut with XbaI/NheI and NheI/SacI, respectively, and inserted into geminiviral vector, resulting in pBYD5-HC. For the light chain of cD5 antibody, the VL gene and constant region of human kappa light chain (CL) was cut with XbaI/AflII and AflII/SacI, respectively, and inserted into geminiviral vector to make pBYD5-LC. Each construct was transformed into Agrobacterium tumefaciens strain GV3101.

### 4.2. Protein Expression, Extraction and Quantification

Six to eight weeks-old of *N. benthamiana* plants were co-infiltrated with an equal amount of two Agrobacterium GV3101 strains containing pBYD5-HC or pBYD5-LC, at a final OD600 of 0.2. For a time-course of expression experiment, leaves were harvested on days-2, -4, -6, -8, and -10 after infiltration. Leaf samples were extracted with 1x PBS (phosphate-buffered saline: 137 mM NaCl, 2.7 mM KCl, 4.3 mM Na_2_HPO_4_, 1.47 mM KH_2_PO_4_) at pH 7.4. The plant-produced cD5 antibody was quantified by ELISA in a 96-well plate (Greiner Bio-One GmbH, Kremsmünster, Austria) coated with 50 μL of anti-human IgG Fc fragment (Abcam, Cambridge, UK) (diluted 1:1000 in 1X PBS) and incubated overnight at 4 °C. The plate was washed three times with 1X PBS-T (1X PBS plus 0.05% Tween-20) and blocked with 5% skim milk in 1X PBS for 2 h at 37 °C. After washing three times with 1X PBS-T, the diluted plant-produced cD5 antibody extracts and human IgG1 kappa isotype antibody (Abcam, Cambridge, UK) were added to the wells and incubated at 37 °C for 2 h. The plate was washed three times with 1X PBS-T and incubated with 50 μL of HRP-conjugated anti-human kappa (The Binding Site, Birmingham, UK) at 37 °C for 1 h. The plate was washed and developed using TMB mixture (Promega, Fitchburg, WI, USA). The reaction was stopped by 1M H_2_SO_4_ and absorbance was measured at 450 nm using a 96-well microplate reader (BMG Labtech, Ortenberg, Germany).

### 4.3. Purification

Infiltrated *N. benthamiana* leaves were extracted with 1 × PBS at pH 7.5 using a blender and centrifuged at 26,000X *g* for 30 min. The supernatant was filtered with a 0.45 μm membrane filter and loaded onto a protein-A bead column. The column was washed with ten-bed volumes of PBS pH 7.5. The bound protein was eluted with 0.1 M glycine, pH 2.7, and rapidly neutralized to pH 7.5 with 1.5 M Tris-HCL pH 8.8. The purified plant-produced cD5 antibody was analyzed using SDS PAGE and western blot.

### 4.4. SDS-PAGE and Western Blot Analysis

In the non-reducing condition, the purified plant-produced cD5 antibody and human IgG control antibody (Abcam, Cambridge, UK) were mixed with the non-reducing loading dye (125 mM Tris-HCl pH 6.8, 12% (*w*/*v*) SDS, 10% (*v*/*v*) glycerol, and 0.001% (*w*/*v*) bromophenol blue) and separated using 6% sodium dodecyl sulfate-polyacrylamide gel electrophoresis (SDS-PAGE). In the reducing condition, the antibodies were mixed with the loading dye (non-reducing loading dye with 22% (*v*/*v*) β-mercaptoethanol) and separated on 6–15% SDS-PAGE. Proteins were stained with InstantBlue (TM) (Expedeon, Cambridge, UK). For western blot analysis, proteins were electrophoretically transferred to a nitrocellulose membrane (Biorad, California, USA) and blocked with 5% skim milk in 1X PBS. The membranes were probed with HRP-conjugated anti-human IgG gamma chain (The Binding Site, Birmingham, UK) or with HRP-conjugated anti-human kappa (The Binding Site, Birmingham, UK) and developed by chemiluminescence using ECL plus detection reagent (Abcam, Cambridge, UK).

### 4.5. Binding Assay

Plant-produced cD5 antibody binding was determined by ELISA assay using a specific peptide. A 96-well plate was coated with 50 μL/well (10 µg/mL) of SP70 peptide (YPTFGEHKQEKDLEY) in PBS buffer and incubated overnight at 4 °C. Next, the plate was washed three times with PBST buffer and blocked with 200 mL of 5% nonfat milk powder in PBS buffer for 2 h at 37 °C. Subsequently, 50 μL/well of diluted cD5 antibody samples in PBS buffer and unrelated human IgG antibody (Abcam, Cambridge, UK), used as a negative control, were added and incubated for 2 h at 37 °C. Later they were incubated with an anti-human kappa chain-HRP antibody (The Binding Site, Birmingham, UK) in PBS buffer for 1 h at 37 °C. The ELISA plate was developed with 50 μL/well of TMB mixture (Promega, Fitchburg, WI, USA) and incubated for 2 min. The reactions were stopped by 50 μL/well of 1 M H_2_SO_4_ and absorbance was measured at 450 nm using a 96-well microplate reader (BMG Labtech, Ortenberg, Germany).

### 4.6. Neutralization Assay

Neutralizing activity of plant-produced cD5 antibody against EV71 clinical strain EV71/G082 was measured using the micro-neutralization assay as described previously [44]. After incubation for three days, cell viability was measured using the CellTiter-Glo 2.0 assay kit (Promega, Fitchburg, WI, USA) following the manufacturer’s protocol. The percentage of neutralization was calculated as follows: 100 × (luminescence of the given sample—luminescence of the virus-only sample)/(luminescence of the cell-only sample—luminescence of the virus-only sample). Half-maximal inhibitory concentration (IC50) of plant-produced cD5 antibody was determined using variable-slope nonlinear regression analysis by GraphPad Prism software.

### 4.7. In Vivo Protection Assay

For evaluating the therapeutic efficacy of plant-produced cD5 mAb, groups of 5-day-old ICR mice were inoculated intraperitoneally (i.p.) with 3.4 × 10^4^ TCID50 (Median Tissue Culture Infectious Dose) of mouse-adapted EV71 strain EV71/MAV-W [45]. One day later, the infected mice were injected i.p. with PBS, plant-produced cD5 antibody (10 µg/g body weight), or murine D5 antibody (10 µg/g body weight). After infection, the mice were monitored daily for 14 days for survival and clinical score. Clinical scores were graded as follows: 0, healthy; 1, reduced mobility; 2, limb weakness; 3, paralysis; and 4, death. All animal studies were approved by the Institional Animal Care and Use Committee at the Institut Pasteur of Shanghai, and the project identification code was 20170324-1. 

### 4.8. Statistical Analysis

All statistical analyses were performed using GraphPad Prism version 5.
